# Association Between PURE Diet Score and Mental Disorders in Patients At‐Risk of Cardiovascular Disease: Findings From the Iran Premature Coronary Artery Disease (IPAD) Study, a Multicenter Cross‐Sectional Study

**DOI:** 10.1002/hsr2.71540

**Published:** 2025-11-23

**Authors:** Noushin Mohammadifard, Ali Emamjomeh, Fahimeh Haghighatdoost, Ehsan Zarepur, Mehdi Abbasi, Ahmadreza Assareh, Mahboobeh Gholipour, Fatemeh Moslemi, Nizal Sarrafzadegan

**Affiliations:** ^1^ Isfahan Cardiovascular Research Center, Cardiovascular Research Institute Isfahan University of Medical Sciences Isfahan Iran; ^2^ Department of Cardiology, School of Medicine Isfahan University of Medical Sciences Isfahan Iran; ^3^ Heart Failure Research Center, Cardiovascular Research Institute Isfahan University of Medical Sciences Isfahan Iran; ^4^ Atherosclerosis Research Center Ahvaz Jundishapur University of Medical Sciences Ahvaz Iran; ^5^ Cardiovascular Diseases Research Center, Department of Cardiology, Heshmat Hospital, School of Medicine Guilan University of Medical Sciences Rasht Iran; ^6^ Iranian Network of Cardiovascular Research (INCVR) Iran

**Keywords:** anxiety, depression, Iran, mental health, PURE diet

## Abstract

**Background and Aims:**

Healthy dietary patterns such as DASH and Mediterranean diets may improve mental well‐being. However, the relationship between the PURE diet—emphasizing moderate consumption of animal foods and whole‐fat dairy—and mental disorders is unclear. This study aimed to investigate the association between the PURE diet score and depression and anxiety in Iranian adults at high risk of cardiovascular disease.

**Methods:**

This multicenter cross‐sectional study included 1120 subjects from nine ethnicities who underwent angiography, with no stenosis detected. Dietary intake was assessed using a validated semi‐quantitative food frequency questionnaire, and the PURE score was calculated based on fruit, vegetables, legumes, nuts, fish, and dairy consumption. Mental health was measured using the Hospital Anxiety and Depression Scale (HADS). Data were analyzed using logistic regression and adjusted for confounders.

**Results:**

Participants' mean (SD) age was 52.08 ± 8.25 years. In the overall population, higher PURE diet scores were significantly associated with lower odds of both depression and anxiety after adjustment for potential confounders (depression: OR = 0.55, 95% CI 0.36–0.86, *p* = 0.03; anxiety: OR = 0.63, 95% CI 0.40–0.99, *p* = 0.01). In sex‐stratified analyzes, these inverse associations were significant among women (depression: OR = 0.56, 95% CI 0.33–0.95, *p* = 0.02; anxiety: OR = 0.52, 95% CI 0.30–0.92, *p* = 0.01), but not among men (depression: OR = 0.49, 95% CI 0.22–1.10, *p* = 0.08; anxiety: OR = 0.78, 95% CI 0.34–1.79, *p* = 0.43).

**Conclusion:**

In this cross‐sectional study, higher adherence to the PURE diet was significantly associated with lower odds of both depression and anxiety, particularly among women. No significant associations were observed among men. Prospective cohort studies and clinical trials are needed to confirm these findings.

## Summary

- Higher adherence to the PURE diet is inversely associated with both depression and anxiety, particularly among women.
- Participants with higher PURE diet scores tended to have better nutrient intake and dietary quality.
- Findings suggest potential mental health benefits of following the PURE diet, especially for women at high cardiovascular risk.

**Abbreviations**
BMI: body mass index
CAD: coronary artery disease
CI: confidence interval
COI: conflict of interest
CVD: cardiovascular disease
DASH: dietary approaches to stop hypertension
FFQ: food frequency questionnaire
HADS: hospital anxiety and depression scale
HEI: healthy eating index
INCVR: Iranian network of cardiovascular research
IPAD: Iran premature coronary artery disease
IPAQ: international physical activity questionnaire
MET: metabolic equivalent of task
OR: Odds ratio
PURE: prospective urban rural epidemiology
RDA: recommended dietary allowance
SPSS: statistical package for the social sciences

## 1 Introduction

Mental health disorders, including depression and anxiety, are prevalent globally and significantly impact quality of life and overall health outcomes [1]. It is estimated that around 25% of people experience depression and 30% suffer from anxiety [2, 3]. Furthermore, there is evidence that individuals with specific health disorders, like cardiovascular disease (CVD), are more prone to the development of mental disorders, in particular depression and anxiety [4]. Indeed, a bidirectional relationship between CVD and mental disorders has been suggested. Therefore, modifying some common risk factors for both conditions may be effective in reducing the burden and progression of disease [4]. For instance, adherence to the American Heart Association's Life's Essential 8 (LE8), an indicator of better cardiovascular health, has been significantly linked to lower odds of depression among emerging adults [5]. In Iran, CVD ranks as the leading cause of death, and therefore CVD and its related conditions take priority over other chronic diseases for research [3].

There is growing evidence that dietary habits may influence both mental and cardiovascular health. Evaluating these habits through dietary scores and patterns offers a structured approach to understanding their effects on health outcomes. Several diet scores, which have initially been developed to improve cardiovascular health, like the Dietary Approaches to Stop Hypertension (DASH), the Mediterranean diet, and the Healthy Eating Index (HEI), are also in a favorable relationship with mental well‐being [6, 7, 8, 9]. Recent evidence further suggests that dietary intake of live microbe‐containing (LMC) foods and engagement in recreational or leisure‐time physical activity (RPA/LTPA) are inversely associated with systemic immune inflammation and depressive symptoms [10, 11]. However, these dietary scores mainly emphasize the consumption of vegetables and healthy fats but restrict the intake of animal foods and whole‐fat dairy. This may not be practical in low‐income countries, where meat and dairy products are often less affordable and consumed in amounts below dietary guidelines [7, 8, 9]. In these regions, economic constraints and cultural norms favor more inclusive and flexible dietary patterns that do not overly restrict food groups like animal products. The Prospective Urban Rural Epidemiology (PURE) diet offers a different approach by focusing on foods to include rather than restricting or avoiding specific food groups. The PURE diet score was developed using data from countries with diverse socioeconomic statuses and, therefore, is more generalizable across various populations. It allows moderate consumption of animal foods and emphasizes fruits, vegetables, nuts, legumes, fish, and whole‐fat dairy, without specifically limiting saturated fats due to ongoing debate about their health effects [12, 13].

To date, a single study specifically investigating the PURE diet score has demonstrated favorable associations with cardiovascular disease and mortality, particularly in low‐ and middle‐income populations [12]. Additionally, broader analyzes of the PURE study, such as its evaluation of macronutrient intake, have provided valuable insights into dietary impacts on cardiovascular diseases [13, 14]. Despite the potential benefits of the PURE diet [12], its correlation with mental health has not been evaluated until now. However, several studies have demonstrated the beneficial effects of its components on mental health status [15, 16]. Earlier dietary indices may not be universally applicable and often have stringent criteria that can be challenging for people who are living in low‐income countries to follow [12]. Therefore, our study aimed to examine the PURE diet score and depression and anxiety in Iranian adults who are at risk of CVD. This population was selected due to the high prevalence of CVD among Iranians and the high burden of mental disorders in this population [3, 17, 18, 19]. In this way, we additionally controlled for the confounding effects of active cardiovascular disease on mental health, allowing us to isolate the impact of diet.

## 2 Methods

### 2.1 Study Design and Population

This multicenter cross‐sectional study included 1120 participants from the Iran Premature Coronary Artery Disease (IPAD) study, a multi‐center case‐control study involving individuals of diverse ethnic backgrounds with recruitment conducted at referral hospitals in Iranian cities and enrollment ongoing through February 2020. The study methodology has been previously described [20]. Briefly, subjects were selected based on the distribution of various ethnicities, including Fars, Azari, Kurd, Arab, Lor, Gilak, Balouch, Qashqaei, and Bakhtiari in 15 cities. Convenience sampling was used to recruit participants for the study. The participants were recruited from reference hospitals in over twelve cities across Iran. This report adheres to the Strengthening the Reporting of Observational Studies in Epidemiology (STROBE) guidelines for cross‐sectional studies [21].

The inclusion criteria for our study were adults with normal coronary angiography to rule out the effect of cardiovascular disease on mental health. Normal coronary angiography was defined as an occlusion of less than 75% in the coronary arteries or less than 50% occlusion in the left main coronary artery. Patients with a history of coronary artery disease (CAD) interventions such as coronary artery bypass surgery, balloon angioplasty, or percutaneous coronary intervention were excluded from the study. Eligible patients were informed about the study, and a written informed consent form was obtained from all participants. The study protocol was reviewed and approved by the Ethics Committee of Isfahan University of Medical Sciences (IR.MUI.REC.1396.2.055), in compliance with the Declaration of Helsinki.

Trained interviewers at hospitals collected demographic and lifestyle information using standardized questionnaires. Physical activity levels were measured with the International Physical Activity Questionnaire (IPAQ) [22]. Height was measured using an elastic meter with participants standing upright without shoes, feet together, and arms hanging naturally at their sides, ensuring the back of the head, shoulder blades, buttocks, and heels touched the vertical board; the measurement was recorded to the nearest 0.1 cm. Weight was measured using a calibrated digital scale with participants wearing light clothing and no shoes, standing still in the center of the scale with weight evenly distributed, and recorded to the nearest 0.1 kg. Waist circumference was measured using an elastic meter at the midpoint between the lower margin of the last palpable rib and the top of the iliac crest and recorded to the nearest 0.1 cm. Body mass index (BMI) was calculated using weight (in kilograms) divided by height in meters squared (m^2^). Participants were also questioned about their medical history and medication use, and their responses were recorded.

### 2.2 Dietary Assessment

A validated semi‐quantitative food frequency questionnaire (FFQ) was used to assess the subjects' eating habits within the last year [23]. The FFQ had a list of 110 food items, and the participants marked how often they consumed each item on a scale of nine options ranging from hardly ever to more than six times a day. The average intake of each food was calculated by combining standard serving sizes with reported frequencies, and daily nutrient intakes were derived using Nutritionist IV software customized for Iranian cuisine.

### 2.3 PURE Diet Score

The PURE diet score evaluates an individual's intake of six food groups: fruit, vegetables, legumes, nuts, fish, and dairy. Each of these six components is assessed individually, with an unweighted score of 0 to 4 based on the quantiles of an individual's intake of the food component. The unweighted sum of the six component scores was then used to calculate the total healthy diet score, ranging from 0 to 24 points. Higher scores indicate a healthier diet [12].

### 2.4 Depression and Anxiety Levels

The Hospital Anxiety and Depression Scale (HADS) was utilized to measure the anxiety and depression levels in participants [24]. This questionnaire comprises 14 questions, with seven for anxiety and seven for depression. Responses were evaluated based on a scale that ranged from 0 to 3. The total score for each section of anxiety and depression could range from 0 to 21. Higher scores are indicative of higher levels of anxiety or depression. Scores below 8 were considered normal, while scores of 8 and above indicated the presence of depression or anxiety.

### 2.5 Statistical Analysis

Pre‐specified analyzes assessed the associations of PURE diet quartiles with the risk of anxiety and depression in the study population. Sex‐stratified analyzes were conducted as exploratory analyzes to assess potential effect modification by sex. Analyzes were performed on available cases with listwise deletion for missing data. Continuous variables are presented as mean ± standard deviation (SD), and categorical variables as number (%). Assumptions for parametric tests were formally evaluated, including normality (Shapiro–Wilk test and visual inspection of residuals) and homogeneity of variances (Levene's test). Means were compared between quartiles of PURE diet scores using one‐way ANOVA. The chi‐square test was used to compare categorical variables across different quartiles. For adjusted mean comparisons, analysis of covariance (ANCOVA) was performed. Effect sizes were also calculated and reported (η² for ANOVA, partial ηp² for ANCOVA, and Cramér's V for chi‐square tests) to quantify the magnitude of associations. To evaluate the association between the PURE diet score and the risk of anxiety and depression, a series of simple and multiple univariate logistic regression models with a logit link function were applied. Referring to previous population‐based studies, several regression models were specified step by step [25, 26]. The initial model examined the unadjusted association. The second model included adjustments for sex, age, and total daily energy intake. Further adjustments were made to the education level and marital status. Subsequent models incorporated additional covariates, including physical activity (MET.h/week), smoking status, and alcohol consumption. In the final model, further adjustments were made to the BMI and city. The study results included crude and adjusted odds ratios (ORs) with a corresponding 95% confidence interval (CI). Quartile 1 (Q1) of the PURE diet score was used as the reference. All analyzes were performed using IBM SPSS Statistics, version 23 (IBM Corp.). Two‐sided tests were applied with a significance level of α = 0.05, and P values were reported following SAMPL guidelines [27].

## 3 Results

The proportion of missing data was low across all variables (< 4%), as shown in Supporting Information Table S1. Table 1 summarizes demographic and lifestyle characteristics of the study participants across the PURE diet score quartiles. No significant differences were observed in age, physical activity levels, BMI, sex distribution, marital status, smoking status, education, or alcohol consumption across quartiles (*p* > 0.05 for all).

**Table 1:** General characteristics of study participants across PURE Diet score quartiles^1^.

	PURE Diet score quartiles				*p* value^2^
	Q1 (*N* = 297)	Q2 (*N* = 226)	Q3 (*N* = 342)	Q4 (*N* = 255)	
Age (years)	52.50 ± 8.55	51.55 ± 8.24	51.88 ± 8.22	51.97 ± 7.58	0.596
Physical activity (MET.h/week)	1758.91 ± 4235.85	1776.91 ± 3521.38	2186.21 ± 5280.14	1845.07 ± 3622.46	0.568
BMI (kg/m²)	29.03 ± 5.81	28.66 ± 4.83	28.64 ± 4.98	28.82 ± 6.06	0.815
Female (*n*, %)	192 (64.6)	144 (63.7)	225 (65.8)	159 (62.4)	0.849
Married (*n*, %)	260 (87.5)	193 (85.4)	313 (91.5)	221 (86.7)	0.112
Current smoker (*n*, %)	47 (15.8)	28 (12.4)	44 (12.9)	35 (13.7)	0.647
Educational category					0.574
Illiterate or Primary (*n*, %)	178 (59.9)	126 (56.0)	186 (54.4)	138 (54.1)	
Secondary and Tertiary (*n*, %)	102 (34.3)	79 (35.1)	123 (36.0)	95 (37.3)	
BSc or A.S (*n*, %)	12 (4.0)	13 (5.8)	24 (7.0)	19 (7.5)	
MSc or PhD (*n*, %)	5 (1.7)	7 (3.1)	9 (2.6)	3 (1.2)	
Alcohol consumption (yes, *n*, %)	9 (3.0)	5 (2.2)	14 (4.1)	10 (3.9)	0.613

^1^ Values are presented as mean ± standard deviation or number (%).

^2^ The *p*‐values were calculated using ANOVA for continuous variables and chi‐square (χ²) tests for categorical variables.

Nutrient intakes varied significantly across the PURE diet score quartiles (Table 2). Higher PURE diet scores were characterized by greater protein and fat intake, lower carbohydrate intake, and increased consumption of fruits, vegetables, and fish, alongside reduced refined grain intake (all *p* < 0.001). Micronutrient intake, including zinc, cobalamin, folate, and pyridoxine, also increased across quartiles (*p* < 0.001 for all).

**Table 2:** Dietary intakes of participants across PURE Diet score quartiles^1^.

	PURE Diet score quartiles				*p* value^2^
	Q1 (*N* = 297)	Q2 (*N* = 226)	Q3 (*N* = 342)	Q4 (*N* = 255)	
Energy (kcal/d)					
Carbohydrate (g/day)	271.28 ± 2.52	249.92 ± 2.86	243.77 ± 2.33	232.18 ± 2.70	< 0.001
Protein (g/day)	79.81 ± 0.90	82.87 ± 1.03	84.91 ± 0.84	87.31 ± 0.97	< 0.001
Fat (g/day)	80.55 ± 1.18	88.02 ± 1.34	90.86 ± 1.09	94.98 ± 1.27	< 0.001
High fat dairy (g/day)	31.91 ± 5.22	54.20 ± 5.93	70.82 ± 4.83	116.46 ± 5.60	< 0.001
Low fat dairy (g/day)	251.77 ± 11.75	250.72 ± 13.34	261.19 ± 10.87	237.64 ± 12.59	0.569
Fruits (g/day)	213.80 ± 5.23	252.04 ± 6.05	297.54 ± 4.93	327.98 ± 5.70	< 0.001
Vegetables (g/day)	279.90 ± 8.18	341.35 ± 9.29	384.83 ± 7.57	398.70 ± 8.77	< 0.001
Red and processed meat (g/day)	23.66 ± 1.71	29.87 ± 1.95	30.49 ± 1.59	34.53 ± 1.84	< 0.001
Fish and shrimp (g/day)	5.78 ± 1.36	15.92 ± 1.54	20.60 ± 1.25	32.68 ± 1.45	< 0.001
Refined grain (g/day)	264.06 ± 7.17	224.08 ± 8.14	217.76 ± 6.63	188.09 ± 7.68	< 0.001
Whole grain (g/day)	189.95 ± 7.20	149.83 ± 8.18	106.99 ± 6.66	80.45 ± 7.72	< 0.001
Legumes (g/day)	45.35 ± 2.98	69.86 ± 3.39	88.56 ± 2.76	93.32 ± 3.20	< 0.001
Fast foods (g/day)	12.27 ± 1.18	11.69 ± 1.33	13.99 ± 1.09	14.01 ± 1.26	0.426
Nuts (g/day)	9.28 ± 0.90	8.50 ± 1.02	10.31 ± 0.83	12.05 ± 0.96	0.060
Iron (mg/day)	13.71 ± 0.17	13.81 ± 0.20	13.83 ± 0.16	13.81 ± 0.19	0.968
Zinc (mg/day)	11.53 ± 0.18	12.40 ± 0.21	13.18 ± 0.17	14.02 ± 0.19	< 0.001
Cobalamin (µg/day)	2.10 ± 0.16	2.98 ± 0.18	3.35 ± 0.15	4.57 ± 0.17	< 0.001
Folate (µg/day)	244.68 ± 3.37	254.06 ± 3.83	263.27 ± 3.12	268.11 ± 3.61	< 0.001
Magnesium (mg/day)	354.72 ± 4.99	347.83 ± 5.66	342.69 ± 4.61	354.35 ± 5.34	0.242

^1^ Values are Mean ± SE. Nutrients were adjusted for age, sex and total energy intake (kcal). Energy intake was adjusted for age and sex.

^2^ From analysis of covariance (ANCOVA) and energy was considered as the absolute amount per day.

Table 3 shows the mean scores of depression and anxiety by PURE diet score quartiles. Participants in the highest quartile consistently had lower depression and anxiety scores compared to those in the lowest quartile, and these trends remained significant across all adjusted models (*p* < 0.05).

**Table 3:** Mean scores of depression and anxiety across PURE Diet score quartiles^1^.

	PURE Diet score quartiles				*p* value^2^
	Q1 (*N* = 297)	Q2 (*N* = 226)	Q3 (*N* = 342)	Q4 (*N* = 255)	
Depression					
Crude	9.19 ± 0.23	8.40 ± 0.27	8.85 ± 0.22	8.13 ± 0.24	0.005
Model 1	9.20 ± 0.23	8.39 ± 0.26	8.81 ± 0.21	8.16 ± 0.25	0.011
Model 2	9.16 ± 0.23	8.36 ± 0.26	8.87 ± 0.21	8.16 ± 0.24	0.011
Model 3	9.17 ± 0.23	8.39 ± 0.26	8.84 ± 0.21	8.16 ± 0.24	0.012
Model 4	9.18 ± 0.23	8.34 ± 0.26	8.83 ± 0.21	8.15 ± 0.24	0.009
Anxiety					
Crude	10.24 ± 0.27	10.03 ± 0.32	10.63 ± 0.25	9.26 ± 0.29	0.010
Model 1	10.21 ± 0.27	10.00 ± 0.31	10.62 ± 0.25	9.34 ± 0.29	0.009
Model 2	10.24 ± 0.27	10.01 ± 0.31	10.63 ± 0.25	9.26 ± 0.29	0.006
Model 3	10.19 ± 0.27	10.00 ± 0.30	10.62 ± 0.25	9.34 ± 029	0.008
Model 4	10.18 ± 0.27	9.95 ± 0.30	10.61 ± 0.25	9.32 ± 0.29	0.008

*Note:* Model 1: Adjusted for sex, age, and total daily energy. Model 2: Additional adjustment for education and marital status. Model 3: Additional adjustment for physical activity, current smoker status, and alcohol consumption. Model 4: Additional adjustment for BMI.

^1^ Values are mean ± standard error.

^2^ Derived from ANOVA in crude and ANCOVA in adjusted models.

Table 4 presents the ORs and 95% CIs for depression and anxiety across the quartiles of the PURE score. For depression, higher PURE scores were associated with significantly lower odds in the fully adjusted model (Model 4: OR = 0.55, 95% CI: 0.36–0.86, *p* = 0.03). This association was slightly weaker in the crude and partially adjusted models but followed the same inverse trend. The OR for anxiety also showed a significant decreasing trend in the fully adjusted model (OR = 0.63, 95% CI: 0.40–0.99, *p* = 0.01), indicating that higher PURE diet scores were linked to lower odds of anxiety after full adjustment.

**Table 4:** Crude and multivariable‐adjusted odds ratio and 95% confidence interval for depression and anxiety by PURE Diet score quartiles.

	PURE Diet score quartiles				*p* value^1^
	Q1 (*N* = 297)	Q2 (*N* = 226)	Q3 (*N* = 342)	Q4 (*N* = 255)	
Depression					
Crude	1 (Reference)	0.85 (0.59, 1.21)	0.89 (0.64, 1.23)	0.71 (0.50, 1.00)	0.083
Model 1	1 (Reference)	0.83 (0.57, 1.20)	0.85 (0.61, 1.19)	0.70 (0.49, 1.00)	0.070
Model 2	1 (Reference)	0.83 (0.57, 1.20)	0.88 (0.63, 1.23)	0.71 (0.49, 1.01)	0.092
Model 3	1 (Reference)	0.83 (0.57, 1.20)	0.87 (0.62, 1.21)	0.70 (0.49, 1.01)	0.080
Model 4	1 (Reference)	0.82 (0.56, 1.19)	0.86 (0.61, 1.21)	0.70 (0.49, 1.01)	0.083
Anxiety					
Crude	1 (Reference)	0.96 (0.66, 1.39)	1.14 (0.81, 1.59)	0.81 (0.57, 1.15)	0.467
Model 1	1 (Reference)	0.95 (0.65, 1.38)	1.13 (0.80, 1.59)	0.82 (0.57, 1.18)	0.511
Model 2	1 (Reference)	0.96 (0.66, 1.40)	1.17 (0.82, 1.65)	0.83 (0.58, 1.20)	0.604
Model 3	1 (Reference)	0.97 (0.66, 1.41)	1.16 (0.82, 1.65)	0.83 (0.58, 1.20)	0.587
Model 4	1 (Reference)	0.96 (0.65, 1.40)	1.16 (0.82, 1.65)	0.82 (0.57, 1.18)	0.545

*Note:* Model 1: Adjusted for sex, age, and total daily energy. Model 2: Additional adjustment for education and marital status. Model 3: Additional adjustment for physical activity, current smoker status, and alcohol consumption. Model 4: Additional adjustment for BMI.

^1^ From Mantel‐Haenszel extension chi‐square test.

Table 5 indicates a sex‐specific association between the PURE score and depression that remained more evident in males only in the crude and partially adjusted models, but became nonsignificant after full adjustment (Model 4: OR = 0.49, 95% CI: 0.22–1.10, *p* = 0.08). However, among females, the fully adjusted model revealed a significant inverse association between PURE diet score and both depression (OR = 0.56, 95% CI: 0.33–0.95, *p* = 0.02) and anxiety (OR = 0.52, 95% CI: 0.30–0.92, *p* = 0.01).

**Table 5:** Sex‐stratified Odds ratios and 95% confidence intervals of depression and anxiety by PURE Diet score quartiles.

	PURE Diet score quartiles				*p* value^1^
	Q1 (*N* = 297)	Q2 (*N* = 226)	Q3 (*N* = 342)	Q4 (*N* = 255)	
Male					
Depression					
Crude	1 (Reference)	0.61 (0.34, 1.09)	0.74 (0.44, 1.26)	0.51 (0.29, 0.90)	0.041
Model 1	1 (Reference)	0.61 (0.34, 1.09)	0.73 (0.43, 1.24)	0.51 (0.29, 0.89)	0.038
Model 2	1 (Reference)	0.61 (0.34, 1.11)	0.75 (0.44, 1.28)	0.51 (0.29, 0.90)	0.045
Model 3	1 (Reference)	0.62 (0.34, 1.12)	0.73 (0.43, 1.26)	0.51 (0.29, 0.90)	0.039
Model 4	1 (Reference)	0.63 (0.34, 1.13)	0.74 (0.43, 1.27)	0.52 (0.29, 0.92)	0.048
Anxiety					
Crude	1 (Reference)	1.03 (0.57, 1.86)	1.44 (0.84, 2.50)	0.86 (0.49, 1.50)	0.932
Model 1	1 (Reference)	1.02 (0.57, 1.85)	1.44 (0.83, 2.50)	0.86 (0.49, 1.52)	0.959
Model 2	1 (Reference)	1.02 (0.57, 1.86)	1.48 (0.85, 2.57)	0.87 (0.50, 1.54)	0.993
Model 3	1 (Reference)	1.05 (0.58, 1.91)	1.47 (0.84, 2.57)	0.87 (0.50, 1.54)	0.985
Model 4	1 (Reference)	1.03 (0.56, 1.87)	1.45 (0.83, 2.54)	0.84 (0.48, 1.50)	0.908
Female					
Depression					
Crude	1 (Reference)	1.05 (0.66, 1.69)	0.98 (0.64, 1.49)	0.89 (0.57, 1.40)	0.596
Model 1	1 (Reference)	1.02 (0.63, 1.65)	0.96 (0.62, 1.46)	0.87 (0.55, 1.38)	0.524
Model 2	1 (Reference)	1.02 (0.63, 1.64)	0.97 (0.63, 1.49)	0.86 (0.54, 1.37)	0.539
Model 3	1 (Reference)	1.04 (0.64, 1.68)	0.98 (0.64, 1.51)	0.87 (0.55, 1.39)	0.560
Model 4	1 (Reference)	1.01 (0.62, 1.64)	0.97 (0.63, 1.49)	0.86 (0.54, 1.37)	0.525
Anxiety					
Crude	1 (Reference)	0.92 (0.56, 1.49)	0.97 (0.63, 1.50)	0.80 (0.50, 1.26)	0.408
Model 1	1 (Reference)	0.92 (0.56, 1.51)	0.99 (0.63, 1.55)	0.80 (0.50, 1.29)	0.467
Model 2	1 (Reference)	0.93 (0.57, 1.52)	1.03 (0.65, 1.61)	0.82 (0.51, 1.33)	0.557
Model 3	1 (Reference)	0.94 (0.57, 1.55)	1.02 (0.65, 1.61)	0.83 (0.51, 1.34)	0.545
Model 4	1 (Reference)	0.94 (0.57, 1.54)	1.02 (0.65, 1.61)	0.82 (0.50, 1.32)	0.527

*Note:* Model 1: Adjusted for age and total daily energy. Model 2: Additional adjustment for education and marital status. Model 3: Additional adjustment for physical activity, current smoker status, and alcohol consumption. Model 4: Additional adjustment for BMI.

^1^ From Mantel‐Haenszel extension chi‐square test.

## 4 Discussion

This study is the first one to examine the association of the PURE diet with mental disorders in people who are at high risk for CVD. We found that higher adherence to the PURE diet was significantly associated with lower odds of both depression and anxiety in this population. Sex‐stratified analyzes further revealed that these inverse associations were significant among women but not among men.

The PURE diet, which encourages a variety of nutrient‐dense foods, is practical in diverse contexts. Unlike restrictive dietary patterns such as the DASH and Mediterranean diets, the PURE diet does not impose strict limits on animal products or dairy but processed foods, offering a wider range of options [6, 28]. This adaptability enhances adherence, particularly in low‐ and middle‐income countries where other dietary patterns may be less feasible [29, 30].

The study found that higher adherence to the PURE diet was associated with lower odds of both depression and anxiety in the overall sample. While there is no study directly investigating the PURE diet in relation to mental health, several studies have examined the components of the PURE diet in this regard [15, 16]. For instance, Oddy et al. linked diets high in processed foods to higher anxiety and depression, while diets rich in fruits and vegetables were associated with better mental health [31]. Akbaraly et al. found that diets rich in vegetables, fruits, and fish were associated with a lower risk of developing depression [32]. Additionally, Kingsbury et al. demonstrated an inverse relationship between fruit and vegetable consumption and depressive symptoms, further underscoring the potential mental health benefits of these diet components [16]. Consistent with our findings, research by Jacka et al. and Oddy et al. suggested that healthier dietary patterns rich in fruits, vegetables, and whole grains were associated with lower odds of anxiety [31, 33]. However, research in a Korean population showed that higher diet quality was linked to lower depression but not anxiety [34].

Prior research has also documented sex differences in diet–mental health relationships. For instance, higher dietary fiber intake (from fruits and vegetables) has been linked to lower depression risk only in women, and nutrients like fiber and sugar have shown different mental health impacts in men versus women [35]. Our findings align with this pattern, suggesting that women in our cohort may have derived greater mental health benefits from the nutrient‐rich PURE diet. Several factors may account for this sex‐specific effect, including biological influences such as hormonal fluctuations—particularly the modulatory role of estrogen on neurotransmitter systems (e.g., serotonin, dopamine) and brain‐derived neurotrophic factor (BDNF), which are critical to mood regulation [36, 37, 38]. Additionally, psychosocial factors like differential exposure to caregiving responsibilities, stress, and social support may influence diet–mental health relationships differently in women [39, 40].

The potential mechanisms behind the beneficial association between healthy dietary patterns and mental disorders might be related to their anti‐inflammatory and antioxidant properties [41]. Their potential to enhance the production of BDNF, an essential neurotransmitter for neuronal survival that is often reduced in individuals with depression. Furthermore, healthy dietary patterns are nutrient‐dense and provide adequate nutrients essential for neurotransmitter synthesis [42, 43]. For instance, zinc regulates brain circuits involved in mood, and folate supports neurotransmitter production, influencing depression [36, 42, 44].

Moreover, despite some differences in examined micronutrient intake in our study across the quartiles of the PURE diet score, the sufficiency or insufficiency of nutrient intake in all four quartiles was similar. For instance, in spite of a significant difference in folate intake across the quartiles of the PURE diet score, subjects in all four groups had an intake lower than the recommended dietary allowance (RDA). Similarly, pyridoxine and zinc intake were higher than the RDA in all four quartiles. These findings suggest that the observed associations are unlikely to be driven by single nutrients alone. Instead, the results are consistent with evidence indicating that overall diet quality and anti‐inflammatory dietary patterns are linked to better mental health outcomes. In contrast, proinflammatory diets are associated with a higher risk of depressive and anxiety symptoms [35, 45]. Moreover, clinical trials and meta‐analyzes have shown that improving overall diet quality can alleviate depressive symptoms. At the same time, micronutrients such as folate and zinc may play supportive yet non‐independent roles in this relationship [46, 47, 48].

This study has several strengths, including a large and ethnically diverse sample of Iranians, which increase the external validity of our findings for Iranian adults. Excluding participants with significant coronary artery disease minimizes confounding factors, offering a clearer link between diet and mental health. The assessment of dietary intakes and mental disorders using validated questionnaires is another strength of this study. However, the cross‐sectional design precludes causal inference, and the association may be bidirectional, as depression could also influence dietary habits. Residual confounding cannot be ruled out despite adjustments for multiple covariates. In particular, unmeasured factors such as socioeconomic status, sleep, comorbidities, medication use, pain, and life stressors may have affected both diet and mental health; Although both the FFQ and HADS are validated instruments, recall bias in dietary reporting and limited sensitivity of HADS for mild anxiety should be acknowledged [49, 50]. Future longitudinal and intervention studies are needed to validate these findings and address potential residual confounding.

## 5 Conclusion

In conclusion, adherence to the PURE diet was inversely associated with depression and anxiety among individuals at high cardiovascular risk, with stronger effects observed in women. Given the cross‐sectional design, these findings should be interpreted with caution, and longitudinal or interventional studies are needed to confirm them.

## Author Contributions

N.M. and N.S. contributed to conceptualization and methodology. N.M., E.Z., A.A., M. Gh., and F.M. were responsible for investigation, data curation, and resources. F.H. conducted formal analysis, validation, and visualization, and contributed to writing – original draft and review and editing. A.E., M.A., and F.H. interpreted the results and drafted the manuscript. N.S. supervised the study. All authors have read and approved the final version of the manuscript. F.H. had full access to all of the data in this study and takes complete responsibility for the integrity of the data and the accuracy of the data analysis.

## Ethics Statement

The study protocol was reviewed and approved by the Ethics Committee of Isfahan University of Medical Sciences (IR.MUI.REC.1396.2.055), in compliance with the Declaration of Helsinki. All participants provided written informed consent before their inclusion in the study.

## Conflicts of Interest

The authors declare no conflicts of interest.

## Transparency Statement

The lead author Fahimeh Haghighatdoost affirms that this manuscript is an honest, accurate, and transparent account of the study being reported; that no important aspects of the study have been omitted; and that any discrepancies from the study as planned (and, if relevant, registered) have been explained.

## Supporting information


**Supplementary Table S1:** Summary of missing data by variable and model.

## Data Availability

The data supporting this study's findings are available from the corresponding author upon reasonable request. Due to privacy and ethical restrictions, these data are not associated with a publicly available repository.
