# Supplementary material for: Association Between PURE Diet Score and Mental Disorders in Patients At‐Risk of Cardiovascular Disease: Findings From the Iran Premature Coronary Artery Disease (IPAD) Study, a Multicenter Cross‐Sectional Study
Source: Health Sci Rep. 2025 Nov 23;8(11):e71540. doi: 10.1002/hsr2.71540 (PMC12641096; doi:10.1002/hsr2.71540)
Supplement: Supplementary file 1 — Supplementary Table S1: Summary of missing data by variable and model. [file HSR2-8-e71540-s001.docx]

**Supplementary Table S1. Summary of missing data by variable and model**

| **Variable / Model** | **Missing (n)** | **Missing (%)** | **Notes** |
| --- | --- | --- | --- |
| **BMI** | 5 | 0.4% | Q1=0, Q2=2, Q3=1, Q4=2 (Table 1) |
| **Depression/Anxiety (HADS)** | 0 | 0.0% | No missing values |
| **Other covariates** (age, sex, marital status, smoking, alcohol, physical activity, education, energy intake) | 0 | 0.0% | Complete data |
| **Logistic regression – Total sample (N=1120)** |  |  | Missingness due to covariates in models |
| Crude model | 29 | 2.6% | Cases excluded due to missing BMI/covariates |
| Model 1 | 29 | 2.6% | Same as crude |
| Model 2 | 30 | 2.7% | Slight increase vs. Model 1 |
| Model 3 | 30 | 2.7% | Same as Model 2 |
| Model 4 | 35 | 3.1% | Highest exclusion |
| **Logistic regression – Sex stratified (Table 5)** |  |  | Excluded cases per model (Male N=700; Female N=420) |
| Male – Crude | 15 | 2.1% | Excluded due to missing BMI/covariates |
| Male – Model 1 | 15 | 2.1% | Same as crude |
| Male – Model 2 | 15 | 2.1% | Same as crude |
| Male – Model 3 | 15 | 2.1% | Same as crude |
| Male – Model 4 | 19 | 2.7% | Slight increase |
| Female – Crude | 14 | 3.3% | Excluded due to missing BMI/covariates |
| Female – Model 1 | 14 | 3.3% | Same as crude |
| Female – Model 2 | 15 | 3.6% | Slight increase |
| Female – Model 3 | 15 | 3.6% | Same as Model 2 |
| Female – Model 4 | 16 | 3.8% | Slight increase |
